# Supplementary material for: Social and healthcare-seeking experiences of people affected with lymphedema in Bangladesh
Source: PLoS Negl Trop Dis. 2025 Aug 12;19(8):e0013384. doi: 10.1371/journal.pntd.0013384 (PMC12342334; doi:10.1371/journal.pntd.0013384)
Supplement: S1 File — (DOCX) [file pntd.0013384.s001.docx]

**Supplementary 1**

**Interview Guideline: Persons with Lymphatic Filariasis**

**Health Care Seeking Behaviour and social experiences of Persons with a history of Lymphatic Filariasis in Bangladesh:**

- Age:
- Sex:
- Education:
- Occupation:
- Marital status:
- Address/location:
- Religion:

1. When did you get diagnosed with LF?

2. When did you first notice the symptoms? i.e., itchy skin (pruritis), abdominal pain, chest pain, muscle pain (myalgias), and/or areas of swelling under the skin. When did you go to seek treatment? Care-seeking delay—in case there is a significant delay, we need to ask:

- Why did you delay taking/seeking treatment?
- Did not realize
- Did not know where to go
- Fear of exclusion/stigma
- Others

3. From where/whom are you taking treatment?

- Doctor/Hospital (Govt., non-govt, chamber, and clinic)
- Traditional healer
- Homeopath
- Others (Please mention/describe in detail)

4. Are you still taking medicines/treatment?

- If not, why? Have you completed it?
- If so, how long have you been taking medicine?

5. Did you face any challenges while seeking treatment?

- If so, please provide us with more details.
- Did you experience any issues while taking your medicine? If so, tell us a little about your experience.
- Did you face more challenges or problems because of your illness/health problem?

If the participant is female, what specific barriers/challenges did they face

6. What are the stigmas related to LF in your society/community? Please describe.

- General perceptions towards the affected person
- Concealing the disease
- Visiting a filariasis-affected person’s house
- Employment of LF affected persons
- LF and marriage
- Social exclusion/isolation

7. How did you/your family members/or your community members react when you/ got to know about your health problem/illness?

- Supportive
- Moderately supportive
- Not at all

8. How did LF impact your life/job?

- Can you carry out your daily activities like you used to before?
- Personal
- Job/work
- Social
- Family roles
- Community participation/social activities

9. In what ways does LF affect /the patient's mental health?

10. How do you think your mental stress and family or society's uncooperativeness have, or could have, affected the progress of your treatment?

11. If the patient is female:

- Because of being a woman/if women have this health problem/illness, do they get more or less support or challenges from family or society? What do you think?

12. What do you think, in which sector (s) actions need to be taken to solve the problems you mentioned earlier?

- Related to social stigma
- Related to the health system
