# Supplementary material for: Social and healthcare-seeking experiences of people affected with lymphedema in Bangladesh
Source: PLoS Negl Trop Dis. 2025 Aug 12;19(8):e0013384. doi: 10.1371/journal.pntd.0013384 (PMC12342334; doi:10.1371/journal.pntd.0013384)
Supplement: S2 File — (DOCX) [file pntd.0013384.s002.docx]

**Supplementary 2_**

**Interview Guideline: Topic guide for NTD stakeholders (Key Informant Interview)**

**Health Care Seeking Behaviour and social experiences of Persons with a history of Lymphatic Filariasis in Bangladesh:**

1. **Background and Experience of the Stakeholder Participant (SP)**

- Can you please tell us about your current role and experience in the field of Neglected Tropical Diseases (NTDs), particularly Filariasis?

1. **Disease Burden and National Program Progress**

- What is the current situation of lymphatic Filariasis (LF) in Bangladesh?
- What major milestones has the program achieved in eliminating Filariasis?

1. **Community Perceptions and Beliefs**

- Have you observed any superstitions or misconceptions regarding Filariasis among the LF patients and in the communities?
- How do these beliefs impact the treatment-seeking behavior?

1. **Health-Seeking Behavior**

- How do patients usually identify that they have Filariasis?
- Who do they approach first for treatment — traditional healers, pharmacies, or healthcare facilities?

1. **Treatment Access and Barriers**

- What kind of treatment is provided to filariasis patients in Bangladesh?
- What are the common barriers faced by patients in accessing treatment?
- Is there a difference in access due to economic status?

1. **Stigma and Discrimination**

- Do filariasis patients face any stigma or discrimination in their communities or within healthcare settings?
- How is the program addressing these issues?

1. **Mental Health and Psychosocial Impact**

- What is the mental health condition of individuals with Filariasis?
- Are there any psychosocial support mechanisms included in the program?

1. **Program Details and Interventions**

- Can you explain the Morbidity Management and Disability Prevention (MMDP) program in detail?
- Is there any involvement of trained mental health professionals in the program?

1. **Training and Capacity Building**

- Are the healthcare workers adequately trained to manage filariasis cases, including physical and psychological care?

1. **Current and Future Plans of the National Program**

- What ongoing support is available for individuals affected by filariasis?
- What are the plans for sustaining filariasis control and integrating other NTDs into the program?
