# Supplementary material for: Social and healthcare-seeking experiences of people affected with lymphedema in Bangladesh
Source: PLoS Negl Trop Dis. 2025 Aug 12;19(8):e0013384. doi: 10.1371/journal.pntd.0013384 (PMC12342334; doi:10.1371/journal.pntd.0013384)
Supplement: S3 File — (DOCX) [file pntd.0013384.s003.docx]

**Supplementary 3: COREQ (Consolidated criteria for Reporting Qualitative research) Checklist**

| Topic | Item No. | Guide Questions/Description | Remark | Reported on Page No. |
| --- | --- | --- | --- | --- |
| Domain 1: Research team and reflexivity | | | | |
| Personal characteristics | | | | |
| Interviewer/facilitator | 1 | Which author/s conducted the interview or focus group? | Lead Authors (KNK and JS) | Methods – 9 |
| Credentials | 2 | What were the researcher’s credentials? E.g. PhD, MD | End of the manuscript | Page 31 |
| Occupation | 3 | What was their occupation at the time of the study? | Lead Author: Associate Scientist | Page 31 |
| Gender | 4 | Was the researcher male or female? | Female: ; Male: | N/A |
| Experience and training | 5 | What experience or training did the researcher have? | The lead author and other co-authors are experienced in conducting both qualitative and quantitative research designs. | Page 9 |
| Relationship with participants | | | | |
| Relationship established | 6 | Was a relationship established prior to study commencement? | No relationship was established with the participants prior to the commencement of the study. | N/A |
| Participant knowledge of the interviewer | 7 | What did the participants know about the researcher? e.g. personal goals, reasons for doing the research | KNK and JS introduced themselves to the participants, stating that both are public health researchers, and described the research team, the purpose of the project, and answered any questions participants may have had about the study and those involved in it. | Page 8 |
| Interviewer characteristics | 8 | What characteristics were reported about the inter viewer/facilitator? e.g. Bias, assumptions, reasons and interests  in the research topic | The interviewers were public health professionals involved in multiple mental health research studies in Bangladesh and also had read the literature related to the study at the beginning of the research | N/A |
| Domain 2: Study design | | | | |
| Theoretical framework | | | | |
| Methodological orientation and Theory | 9 | What methodological orientation was stated to underpin the study? e.g.  grounded theory, discourse analysis, ethnography, phenomenology, content analysis | Inductive and deductive thematic analysis | Methods – 10 |
| Participant selection | | | | |
| Sampling | 10 | How were participants selected? e.g. purposive, convenience, consecutive, snowball | Purposive selection | Methods – 9 |
| Method of approach | 11 | How were participants approached? e.g. face-to-face, telephone, mail, email | Face-to-face, telephone, Zoom, and email | Methods – 9 |
| Sample size | 12 | How many participants were in the study? | Total Number of participants: 28 | Result - 11 |
| Non-participation | 13 | How many people refused to participate or dropped out? Reasons? | Out of 52 on the NFEP patient list, 8 have moved out of the area, 14 were unwell and 10 could not establish a meaningful conversation. | Result - 11 |
| Setting | | | | |
| Setting of data collection | 14 | Where was the data collected? e.g. home, clinic, workplace | Online meeting and face-to-face interview in Thakurgaon (TKG) and Panchagarh | Methods – 8 |
| Presence of nonparticipants | 15 | Was anyone else present besides the participants and researchers? | We asked participants to be in a private place that they preferred, where they would not be disturbed. However, in some instances, we could not prevent the caregiver of persons with LF from occasionally disturbing the interview. | N/A |
| Description of sample | 16 | What are the important characteristics of the sample? e.g. demographic data, date | Reported in the results section | Results – 11 |
| Data collection | | | | |
| Interview guide | 17 | Were questions, prompts, guides provided by the authors? Was it pilot tested? | All questions are provided in the Additional Files. | S1 File  S2 File |
| Repeat interviews | 18 | Were repeat interviews carried out? If yes, how many? | There was no repeat interview. | N/A |
| Audio/visual recording | 19 | Did the research use audio or visual recording to collect the data? | All the interviews, both face-to-face and online, were audio recorded | Methods –9 |
| Field notes | 20 | Were field notes made during and/or after the interview or focus group? | Other authors took notes during interviews | N/A |
| Duration | 21 | What was the duration of the interviews or focus group? | The interviews lasted for  30-40 minutes | Methods – 9 |
| Data saturation | 22 | Was data saturation discussed? | Data saturation was reached when no new data emerged from the interviews. | Result-11 |
| Transcripts returned | 23 | Were transcripts returned to participants for comment and/or correction? | No | N/A |
| Domain 3: analysis and findings | | | | |
| Data analysis | | | | |
| Number of data coders | 24 | How many data coders coded the data? | Four | Methods -10 |
| Description of the coding tree | 25 | Did authors provide a description of the coding tree? | No | N/A |
| Derivation of themes | 26 | Were themes identified in advance or derived from the data? | Derived from the data | Methods -10 |
| Software | 27 | What software, if applicable, was used to manage the data? | No | N/A |
| Participant checking | 28 | Did participants provide feedback on the findings? | No | N/A |
| Reporting | | | | |
| Quotations presented | 29 | Were participant quotations presented to illustrate the themes/findings?  Was each quotation identified? e.g. participant number | Yes | Results – 11- 27 |
| Data and findings consistent | 30 | Was there consistency between the data presented and the findings? | Yes | N/A |
| Clarity of major themes | 31 | Were major themes clearly presented in the findings? | Yes | Results – 11-27 |
| Clarity of minor themes | 32 | Is there a description of diverse cases or discussion of minor themes? | Yes | Results – 11-27 |
