# Supplementary material for: Social and healthcare-seeking experiences of people affected with lymphedema in Bangladesh
Source: PLoS Negl Trop Dis. 2025 Aug 12;19(8):e0013384. doi: 10.1371/journal.pntd.0013384 (PMC12342334; doi:10.1371/journal.pntd.0013384)
Supplement: S4 File — (DOCX) [file pntd.0013384.s004.docx]

**Supplementary 4: Thematic Codebook**

| **Theme** | Subtheme | Codes |
| --- | --- | --- |
| 1. **Knowledge and Social Experiences about LF** | - 1. Knowledge about LF | - Filariasis was locally known as “god rog”. - Most participants reported being unaware of the disease when they were affected. - Perceived reasons: contagious, consuming expired or allergic foods, the almighty had punished him for the wrong deed of his ancestors. - The condition could never be cured. |
|  | - 1. Social experiences of LF | - Received uncomfortable stares and avoidance from their community. - Such behaviour caused them to feel shame and embarrassment. - They received enough support from their family members, facing no stigma. - Participants referred to their sons, daughters-in-law, husbands, and fathers as major caregivers. - They all mentioned needing assistance with daily activities such as cooking, eating, using the toilet, accessing healthcare services, and maintaining self-care related to lymphedema. |
|  | - 1. Mental health difficulties | - Felt overwhelmed due to their chronic health conditions. - Expressed sadness (Mon Kharap) due to their struggles with daily life activities and social engagement. - The stigmatized attitudes from the community and frequent discomfort and pain caused low mood and distress. - Concerned about the future of their families. |
| 1. **Health care-seeking pattern** | Delay in care seeking | - Most of them sought treatment after 2-5 years of symptom presentation. - Received conventional healthcare services from a proximity - After the diagnosis of LF by local community clinic physicians. - Received advanced treatment from Sadar Hospital - Some participants (n = 3) sought services from private doctors; only two mentioned consulting traditional healers. - Took free-of-cost medicines - Low therapeutic adherence - Had to take other medicines for other health issues - Low satisfaction with the LF treatment, as Lymphedema was not cured - Received different types of hygiene supplies and self-care training from their community clinics. |
| 1. **Theme: Barriers to seeking healthcare services and perceived recommendations:** |  | - Lack of knowledge about LF: Symptoms were overlooked - Financial complications - Believed it was unnecessary to spend money on an incurable illness. - Distance was perceived as another barrier to accessing healthcare services by persons with LF. - Participants desired to visit tertiary care facilities that were distant from their residences. - Mobility restriction due to the condition |
| 1. **Perceived recommendations of the participants:** |  | - Financial assistance is needed, as they cannot engage in economic sectors. - Job opportunities - Free medical care - Community-based awareness programs and rehabilitation services. - Increased accessibility to healthcare services for persons with LF. - Mental health counselling services are available at their nearest healthcare centres for their psychological distress. |
