# Supplementary material for: Social and healthcare-seeking experiences of people affected with lymphedema in Bangladesh
Source: PLoS Negl Trop Dis. 2025 Aug 12;19(8):e0013384. doi: 10.1371/journal.pntd.0013384 (PMC12342334; doi:10.1371/journal.pntd.0013384)
Supplement: Attachment — Submitted filename: Response to the reviewers.docx [file pntd.0013384.s005.docx]

**Response to the reviewers**

1) Where possible, we encourage authors to provide relevant data underlying their research with their submission in order to facilitate editorial and peer review, even if the data may be restricted from publication or made private by a data repository prior to article acceptance. Many repositories permit private access for review purposes, and have policies for public release at publication. For data concerning human research subjects, please anonymize the data as appropriate and follow pertinent legal and ethical protocols prior to sharing with editors and reviewers. If you are unable to share any pertinent data at the time of editorial and peer review, please kindly let the journal office know.

Response: Thank you for the concern. Please find our response in the editorial manager as well as given below.

The full data of this study is not available in a public repository due to ethical restrictions. The data is really sensitive since the participants are vulnerable in terms of their health condition and belong from marginalised populations. The study involved in depth and key informant interviews with participants providing written and verbal consent, additionally the data are already anonymised to protect the participants' confidentiality. Moreover, the research team ensured that all the data to be stored in a secure place where only the core research team can access. The study has been conducted with the support of Directorate General of Health Services of the Ministry of Health and Family Welfare of Bangladesh government and Public Health Foundation, Bangladesh. However, in case of a request of any pertinent data during peer review, we will request you to contact Professor Sharmeen Yasmeen, Chair of the PHFBD-ERC, Public Health Foundation, Bangladesh ([sharmeenbmc@yahoo.com](mailto:sharmeenbmc@yahoo.com)).

We have included this paragraph in the Data Availability section at page number 32.

2. We see that your study includes live participants, but you have not included an Ethics Statement. Please update your manuscript file to include an Ethics Statement subsection to your Materials and Methods section. It should include:

i) A statement that formal consent was obtained (must state whether verbal/written) OR the reason consent was not obtained (e.g. anonymity)

Response: Thank you very much for the comment. We previously provided our ethics statement in the manuscript. However, upon your request we have re-edited the section. Please find the following paragraph in page number: 10

This study protocol was reviewed and approved by the Institutional Review Board (IRB) of Public Health Foundation, Bangladesh Ethical Review Committee (PHFBD-ERC) (PHFBD-ERC-SF03/2024). The authors adhered to the National Disability Authority (NDA) and the Convention on the Rights of Persons with Disabilities (CRPD) throughout the process. Before the interview, informed consent was obtained by the interviewers, making it clear that participation was voluntary. Written and verbal consents both were obtained from the participants with LF. Moreover, if persons with LF had any form of disability in their upper limbs we obtained verbal consents. Additionally, NTD stakeholders provided verbal consents during their online interviews. All the participants were informed about the study's objectives and purpose in an easy language (Bangla) before data collection. Participants were granted complete control to halt their interviews or discussions at any point in time. They were also requested to provide consent for audio recording of the interviews. Additionally, psychosocial support sessions and referrals to the National Institute of Mental Health (NIMH: Tertiary level public institute for mental health) were offered to persons affected by LF.
