# Supplementary material for: Social and healthcare-seeking experiences of people affected with lymphedema in Bangladesh
Source: PLoS Negl Trop Dis. 2025 Aug 12;19(8):e0013384. doi: 10.1371/journal.pntd.0013384 (PMC12342334; doi:10.1371/journal.pntd.0013384)
Supplement: Attachment — Submitted filename: Response to Reviewers.docx [file pntd.0013384.s006.docx]

**Ref: PNTD-D-25-00355**

**Social and healthcare-seeking experiences of people affected with lymphedema in Bangladesh**

Section Editor

PLOS Neglected Tropical Diseases

We are pleased to have an opportunity to revise our manuscript entitled ***"Social and healthcare-seeking experiences of people affected with lymphedema in Bangladesh."*** We have carefully considered the reviewers' suggestions and adjusted accordingly, as indicated by the tracked changes. We have extensively worked on our introduction, methodology, analysis, results, and discussion sections. In this letter, we have addressed each reviewer's comments and provided our response. For your convenience:

- Reviewers' comments are formatted in **bold**,
- Authors' responses have regular formatting,
- Direct statements from the article are *italicised*.

We found the reviewers' comments highly constructive and appreciate their feedback. Addressing their suggestions has significantly enhanced the quality of our article.

Kind regards,

**Dr. Kamrun Nahar Koly**

**Corresponding author**

**Journal Requirements**

1) **Please ensure that the CRediT author contributions listed for every co-author are completed accurately and in full.**

**At this stage, the following Authors/Authors require contributions: Kamrun Nahar Koly, Jobaida Saba, Zinnatun Nessa, Farhana Rahman Luba, Irin Hossain, M.M. Aktaruzzaman, Md. Golam Rabbani, Milena Simic, Laura Dean, and Julian Eaton. Please ensure that the full contributions of each author are acknowledged in the "Add/Edit/Remove Authors" section of our submission form.**

**The list of** **CRediT author contributions may be found here: https://journals.plos.org/plosntds/s/authorship#loc-author-contributions**

**Response:** Thanks for the comment. We updated the CrediT author contribution section.

**2) Tables should not be uploaded as individual files. Please remove the table files from the online submission form. Tables should be included in your manuscript file as editable, cell-based objects. For more information about how to format tables, see our guidelines:**

[**https://journals.plos.org/plosntds/s/tables**](https://journals.plos.org/plosntds/s/tables)

**Response:** Thanks for the comment. We removed tables from the individual files. All are now provided in the manuscript file.

3) **PLOS journals require authors to make all data necessary to replicate their study's findings publicly available without restriction at the time of publication. When specific legal or ethical restrictions prohibit public sharing of a data set, authors must indicate how others may obtain access to the data.**

**PLOS strongly recommends sharing data in a repository whenever possible. Data repositories improve discoverability and accessibility, ensure long-term preservation, and lead to increased attention for the research.**

**Please state where the data is stored and how readers can access it. Please also provide contact information for a data access committee, ethics committee, or other institutional body to which data requests may be sent.**

**Note: The point of contact cannot be an individual. It needs to be an institutional or ethics board email contact.**

**Please update your Data Availability statement in the online submission form after redrafting it more formally and concisely as this will be published in the paper.**

**Response:** Thank you for pointing out this important expectation. Due to the sensitivity of the study data, it has not been made publicly available. This is primarily because we feel that despite anonymity being integral to the way data are collected and stored, there is a risk (felt by the participants) that their identity may be recognised from the information shared. We have provided our data availability statement in this document and also in the manuscript. Please find the following paragraph on page number 37.

*"The data for this study is not available in a public repository due to ethical restrictions. The data is very sensitive since the participants are marginalised due to their health conditions and associated disabilities. The study involved in-depth and key informant interviews with participants. Despite the data being anonymised to protect the participants' confidentiality and the fact that consent was obtained after describing measures to protect identity, there remains a concern that people might be identifiable due to the details of the interviews. The study was conducted with the support of the Directorate General of Health Services of the Ministry of Health and Family Welfare, Bangladesh, and the Public Health Foundation, Bangladesh, which holds the data. In case of a request for access to pertinent data, please contact Professor Sharmeen Yasmeen, Chair of the PHFBD-ERC, Public Health Foundation, Bangladesh (irb@publichealthfoundation.org.bd)."*

**Reviewers' Comments to Author**

1. **Reviewer #1:**

- **Lack of a healthy control group or comparison with other chronic diseases: Without a control group (e.g., individuals with diabetes or chronic venous insufficiency), it is impossible to determine whether the impact of lymphedema differs from other chronic conditions.**
- **Additionally, the sample size is too small (only 20 patients and 8 healthcare policymakers), limiting the ability to capture variations across gender, economic status, and disease duration.**

**Response:**

(i) Thank you for this point regarding the absence of a healthy control group or comparison with other long-term conditions and small sample size concerns. This qualitative study was designed to explore the lived experiences of individuals affected by lymphatic filariasis (LF) and related healthcare stakeholders in low-income settings. Since this is the first-ever study to describe the health and social experiences of persons with LF in Bangladesh, we focused on gathering in-depth insights from the participants, which would have been difficult to obtain through quantitative analysis or comparative study designs. This study discussed the insights from the people affected with LF, a highly stigmatized condition, and they are often subjected to experience discrimination based on their visible lesions or disabilities, which is not the same in nature for people with diabetes or other chronic diseases. Additionally, the study's objective was not to compare but to highlight their lived experiences. However, we have added a note in the discussion section acknowledging the study's limited scope. Please find the following lines on page 38.

*While this study provided insights into the experience of people living with LF, the lack of an appropriate control group, either including healthy controls or people with other chronic debilitating diseases, may have reduced generalizability. This qualitative study was designed to explore the lived experiences of individuals affected by lymphatic filariasis (LF) and related healthcare stakeholders in low-income settings. Therefore, we focused on gathering in-depth insights of the participants, which would have been difficult through quantitative analysis or comparative study. We found that people affected with LF were highly stigmatised, and often subjected to discrimination based on their visible lesions or disabilities. Some aspects of their experience may be relevant but are unlikely to be easily applicable to people with other chronic debilitating diseases.* *Future large-scale studies focusing on quantitative data should be conducted to validate these findings and explore the social and health-care-seeking experiences of PWLF. Moreover, we intended to gather the lived experiences of LF-affected Bangladeshi individuals in this study, which was not presented in any other studies that may have introduced bias. Therefore, future studies should integrate objective measures or adopt a mixed-methods approach to gain a better understanding.*

***(ii)*** Additionally, in a qualitative study, the sample size depends on reaching saturation. After interviewing 20 participants, we observed that no new insights were emerging, indicating that saturation had been reached. However, the sample population included both male and female participants from two different endemic areas in Bangladesh. There was also variation in disease durations and disability levels. Additionally, we aimed to depict the thematic saturation of the findings gained within the current sample population. Please find the following lines on page 38:

*Moreover, we intended to gather the lived experiences of LF-affected Bangladeshi individuals in this study, which was not presented in any other studies that may have introduced bias. Therefore, future studies should integrate objective measures or adopt a mixed-methods approach to gain a better understanding.*

2. **(i) Overreliance on patient self-reports without objective validation: The study does not incorporate objective indicators (e.g., medical record reviews, treatment costs) to verify the accuracy of patient-reported experiences.**

**(ii) It is essential to integrate quantitative data (e.g., medical records, prescription data, healthcare expenses) and include a broader range of stakeholders, such as community health workers, herbal medicine practitioners, and policymakers.**

**Response: (i)** Thanks for the comment. We agree that integrating objective measures, such as medical record reviews, treatment costs, and prescription data, would provide a more comprehensive picture and enrich the introduction and discussion. We considered this but decided not to pursue it for the following three reasons:

1. Past studies demonstrated that medical records are limited in themselves, as only a minority of affected individuals seeks care from medical practitioners, resorting instead to traditional healers ((1)) who don't keep medical records. Surveying traditional healers (and indeed training them in LF recognition, referral and self-care) would indeed be a valuable venue for further research (and response).
2. As mentioned, we were provided with medical records through the National Filariasis Elimination Program, which included free care consisting of diagnosis and free prescriptions (Line 150, Page 7). This information was used to provide details on the time since diagnosis (duration of the disease) and the treatment-seeking gap (diagnosed but not on treatment). In terms of follow-up healthcare costs, we examined the experiences of individuals living with LF and their financial challenges in accessing and paying for treatment.
3. This is an exploratory qualitative study, intentionally focusing on the lived experiences of individuals with LF in Bangladesh, as well as their daily life accounts, and on service providers' perceptions of the overall care-seeking aspects of individuals with LF. In-depth interviews are designed to capture subjective experiences as a means of understanding the needs, utilisation of available care, and gaps in services to help healthcare providers and policymakers consider how to close such gaps. We agree that interviewing healthcare providers, researchers, policymakers, and other stakeholders adds perspectives on service providers' views, aspirations, and limitations in enhancing care for people affected by LF. We indeed included an analysis of interviews with such stakeholders– please see Table 2 (where we have now added the information on the type of each interviewed stakeholder) on page 14, line 291. We fully agree that the perspectives of informal care providers (traditional healers, Unani, or herbal medicine practitioners) would provide further insights into care-seeking (Line number 765, Page 40). We acknowledged this in our limitation section.

We have interviewed different stakeholders who were national programme consultants of a local non-profit organisation, research consultants of DGHS, representatives of an international non-government organisation, and researchers (Table 2) and please check pages 12-14:

(ii) As this was an exploratory qualitative design, we relied on in-depth interviews to capture subjective experiences. We have acknowledged this limitation and noted the importance of mixed-methods approaches in future work. Please refer to the strengths and limitations section on page 38, lines 740-770.

*"R**esponses of persons with LF may have been influenced by the stigma associated with the condition and societal acceptability, causing social desirability bias, which was controlled by ensuring anonymity and confidentiality. Regarding the dropout rate of 32 out of 52 participants, a nonresponse bias may have been introduced due to systematic differences in terms of demographic characteristics, health status, or other relevant factors.*

3. **(i) Lack of a structured thematic analysis framework: While the study employs thematic analysis, it fails to define each theme explicitly, leading to largely descriptive summaries without a detailed coding framework (codebook).**

**(ii) The study does not sufficiently explore how the disease influences patients' decision-making processes.**

**(iii) The analysis should align interview data with theoretical frameworks such as the Health Belief Model (HBM) or the Social Ecological Model (SEM) to enhance interpretability.**

***|*Response:** (i) We employed thematic analysis but recognise that we could have explained the analysis process in more detail. We have now included the thematic analysis of the findings in Fig. 1 and the coding framework in Supplementary 4.

(ii) Our study examined the social and care-seeking experiences of individuals with lymphedema. In our results section, we described the pattern of healthcare-seeking experiences and the barriers affecting care-seeking behaviour, which included individual, societal, and systemic factors. Please see updated Theme 4 on pages 21-27.

(iii) Thanks for the valuable suggestion. After exploring the SEM model, we understood our findings align with this theory. Therefore, we included the SEM model in our methodology and discussion section.

**Please find the following lines in the method section on page 7, line number 156-180.**

*Two types of interview guidelines were developed, which were informed by the literature and socio-ecological model (SEM) of healthcare-seeking behaviour, leading to the following themes related to knowledge gaps, misconceptions, incurability beliefs (2); emotional distress, and mental health impact (3); gender-specific challenges, family support, hiding symptoms (4); community level stigma, isolation, avoidance of gatherings (5); barriers to access: distance, cost, medication shortages (6); and need for financial assistance, disability recognition, systemic neglect(7). We attempted to identify various factors that influence the care-seeking pattern of individuals with LF. Our interview guides (Supplementary 2) included questions about participants' understanding of LF (23), their experiences of stigma after being diagnosed with LF (19), treatment-seeking pathways, the impact of LF on personal, professional, and social life, and their knowledge about mental health related to NTDs. The interview guideline for persons with LF also included questions about their understanding of available services and perceived challenges LF faces when seeking and utilising healthcare services. On the other hand, the guides for NTD stakeholders (Supplementary 3) addressed the current situation of LF burden in Bangladesh, community perceptions and beliefs about LF, health-seeking behaviour, treatment access and barriers, mental health and psychosocial impact of LF, and programmatic support for individuals with LF.*

***Please find the following line in discussion section: page 35, Line 835***

*Regarding the barriers to seeking healthcare among persons affected with LF, our study findings align with the Social Ecological Model (SEM) (8), a theoretical framework that provides a comprehensive understanding of how different factors interplay and influence people's healthcare-seeking behaviour and health outcomes. Low health literacy about LF, overwhelming emotional responses, and distress about LF were common at the individual level, and such perceptions resulted in delayed care seeking. Interpersonal connections, defined by caregivers' involvement and family support, played a vital role in ensuring access to care. However, the gender disparity and social stigma affected females particularly and resulted in reduced help-seeking tendencies. On the other hand, the dominant role of community-level stigmas and misconceptions of LF pushed people with LF towards experiences of judgement and social withdrawal, further reinforcing delayed care seeking. At a system level, structural barriers marked by long distances, low transportation facilities, and unavailability of mental health services reduced people's dependency on in the formal health system and encouraged reliance on informal care. Finally, at the policy level, participants expressed concerns about more substantial governmental efforts, such as providing financial assistance or accessible rehabilitative services. Therefore, applying SEM underscores the need for integrated efforts to improve the overall care pathway.*

4. **Limited quantitative analysis of social and healthcare barriers: The study does not quantify associations between key variables (e.g., social discrimination vs. healthcare delays). It also does not use crosstab analysis to compare gender, income level, and geographic disparities in healthcare accessibility. To strengthen the findings, quantitative analyses (e.g., chi-square tests, logistic regression) should be incorporated to explore the influence of social factors on healthcare-seeking behaviour.**

**Response:** Thank you for this observation. As a qualitative study, we aimed to explore contextual narratives. We have noted the limitations and that future studies should incorporate quantitative approaches.

5. **This study explores the social and healthcare-seeking experiences of individuals with lymphedema in Bangladesh, using qualitative research methods (semi-structured interviews and key informant interviews). The study analyses the challenges faced by patients in terms of disease awareness, social exclusion, economic burden, healthcare accessibility, and mental health. However, the study has several limitations.**

**Response:** Thank you for your insights. We have addressed the limitations and appropriate future implications.

**Reviewer #2: Introduction:**

6. **line 81 and 82- when describing Culex and Anopheles mosquitos, is this urban/semiurban and rural areas of Bangladesh or globally or where? For Aedes its clear its specific to the Pacific islands.**

**Response:** Thank you, now we deleted the lines from the manuscript.

7. **Methods: lines 148-150- restructure sentence. A topic guide with open ended questions is the tool for a semi-structured interview so saying 'as well as a topic guide...' does not make sense.**

**Response:** Thank you. We have restructured lines 158 for clarity. Please find the following line in page 7:

*"A semi-structured interview format, allowing the addition of open-ended responses, was used for in-depth interviews (IDI) with people with LF and Key Informant Interviews (KII) with stakeholders."*

8. **Line 150-156- Make it clearer the topics covered for each of the two groups of pts.**

**Response:** Thank your feedback. We included the topics covered for each group (please refer to the previous response to question 3). Please find amendments in line number 158-186 -163 in page number 7.

9. **line 161- 'felt well enough to participate'- what does this mean? Is this well enough physically, emotionally? Does it mean 'agree to participate' which is more common wording used around inclusions/exclusion criteria.**

**Response:** Thank you for your observation. We clarified that "felt well enough" refers to both physical and emotional readiness. We have revised it to "agreed and were able to participate." Please see the revised line 199-201, page 9. .

10. **What was the eligibility criteria for KIIs?**

**Response:** We included the eligibility criteria for KIIs in page 9, line number 194-203.

*"Potential adult participants from Thakurgaon and Panchagarh were recruited from the* *NFEP (National Filariasis Elimination Program) database of persons with LF. We determined the eligibility of the participants based on two criteria: a. the patient still resided in the study area, and b. agreed and was able to participate. The interviewer communicated with the persons with LF over the phone an initial engagement. KII participants were purposively selected from the lead author's relevant professional NTD networks based on their involvement in NTD and LF-related health service provision, research, and healthcare intervention implementation programmes for at least 2 years."*

11. **How did you consent people who were illiterate?**

**Response:** Thanks for the question. We described the consent procedure for individuals who could not read or write in the ethics statement (lines 252-263). Please find the following lines in the manuscript.

*"Written and verbal consents both were obtained from the participants with LF. Moreover, if persons with LF had any form of disability in their upper limbs or were unable to read and write, we obtained verbal consent. Also, we obtained thumbprints in the presence of a witness (family member).” "*

12. **Provide the KII guide as a supplementary file.**

**Response:** The KII topic guide has been included as a supplementary file (Supplementary 3)

13. **Overall review and revise methods to make clear how the two different methods (IDIs and KII) were done.**

**Response:** Thank you for the suggestion. We reviewed and updated the method section in the manuscript and provided clear information about both interview methods. Please see pages 7-12.

14. **Line 219-220- reference the previous research that is mentioned. But also isn't saturation something you determine? Did you reach saturation?**

**Response:** Thank you for your constructive feedback. After interviewing 20 participants (less than our original estimate of the number needed), we observed that no new insights emerged.

We ceased data collection after interviewing 20 participants from Thakurgaon and Panchagarh, as well as 8 stakeholders, when thematic saturation was reached.

We have clarified this point within the revised manuscript on page 9:

*Initially, the intention was to include three districts (Thakurgaon, Panchagarh, and Nilphamari), but data collection was guided by the principle of thematic saturation. After conducting 20 interviews across Thakurgaon and Panchagarh, as well as 8 stakeholder interviews, thematic saturation was achieved, with no new significant themes emerging. Consequently, we concluded that data collection at that point and interviews were not conducted in Nilphamari or with additional stakeholders, as further data were deemed unlikely to yield novel insights relevant to the study objectives.*

15. **Table 1- age and duration of condition need units**

**Response*:*** Thank you for the observation. The table has been revised accordingly. Please see Table 1 in page 13.

16. **when using quotes, usually pt IDs are provided with the profile of pts. There is also a table of pts, so one can then easily check that table to see other characteristics of the pt that said that quote if the ID is provided.**

**Response:** Thank you so much for your input. Patient IDs have been included in both tables for better clarification.

17. **line 234- "knowledge of LF among affected individuals and caregivers". Caregivers are not mentioned as participants. if caregivers were interviewed, this needs to be covered in the methods.**

**Response:** Thank you for your insight. It was a typing mistake and caregivers were not directly interviewed; we have revised the text to clarify that caregiver experiences were reported by patients, not elicited directly.

18. **Theme 2- did your data speak to the drivers of stigma? Often with skin conditions, its physical appearance but what role(s) do knowledge, local beliefs, fear for example also play?**

**Response:** Thank you for the insight. Our study identified the drivers of stigma as appearance, community beliefs, and misconceptions. We have discussed these in Theme 2: Social Experiences of Living with Lymphatic Filariasis (LF). Please see the result on page 17.

We updated our discussion section with the following lines. Please find the following lines about the drivers of stigma, including appearance, community beliefs, and misconceptions. Based on your suggestion we also have included this in the discussion, please see page 32:

*"Our data on participants' social experiences provided valuable insights regarding the drivers of stigma experienced by individuals with LF. While the physical manifestations of LF, particularly lymphedema, undoubtedly contribute to stigma due to altered appearance and looks, participants' narratives suggested that a lack of accurate knowledge about LF within communities, coupled with fear regarding its potential transmission, contributed to social withdrawal and isolation among persons with LF. Furthermore, the internalisation of local beliefs, such as the perception of LF as a form of divine punishment, seemed to influence participants' self-worth and acceptance of their condition, potentially exacerbating internalised stigma. Thus, our data suggest that while physical appearance is a factor, the social experiences of individuals with LF are shaped by a complex interplay of limited knowledge, fear of contagion, and cultural and religious beliefs, all of which contribute to the overall burden of stigma. Consistent with our findings, several studies also identify fear of contagion and the inability to fulfil societal roles as significant contributors to stigma among individuals with LF (9-13)."*

19. **line 400- Can you provide more of an explanation around the costs incurred for LF treatment in this context. This comes out again in line 500 on solutions, 'free medicines'. But the readers need more of an understanding of the costs to patients and what is provided by the health system.**

**Response:** We have updated both sections. Please find the following amended sections in the manuscript:

Page 24: "*Participants highlighted that besides using free-of-cost medicine and hygiene supplies, they had to bear out-of-pocket payments for additional medication, analgesics, or skin-care supplies from local drug shops and pharmacies, which were burdensome for the individuals of LF since most of them live in poverty."*

Page 26: "*Distance was also perceived as another barrier to accessing healthcare services by persons with LF. Although participants mentioned receiving support from local community clinics, participants who needed specialised treatment had to visit tertiary care facilities far from their homes. Such transportation expenses often posed additional out-of-pocket expenditures and acted as a barrier."*

20. **line 412-413- hiding symptoms is not a stigmatising attitude but a coping mechanism**

**Response:** Thank you for the input. We corrected the line about "hiding symptoms" to reflect it as a coping mechanism in page 25.

*"NTD stakeholders highlighted that internalised stigma impacted healthcare-seeking patterns. Coping mechanisms to respond to stigmatising attitudes, such as the tendency to hide the symptoms or self-isolate, often lead to misdiagnosis of the condition and treatment delay.*

21. **was there any stigma from health workers that may be a barrier to seeking care?**

**Response:** There was no stigma from the health workers that was mentioned by the participants. Hence, they were not addressed, but we recognise this is a major finding in other studies and a surprise that we did not find this. This was addressed on page number 35, line 691.

*Moreover, some studies have also discussed how experiencing stigmatised attitudes from healthcare providers impacts the healthcare-seeking attitudes of persons with LF. We were surprised that our participants did not recall facing any stigma from healthcare providers (19, 68). Because such interactions are often common in particular settings where healthcare providers have limited LF-specific training, they can result in neglect, verbal judgment, or a lack of empathy. This situation reflected the* ***recent efforts in the NFEP program of the Bangladesh Government to improve health service delivery****. However this may also indicate that patients have low hopes of care and thus did not translate neglecting behaviour as stigma. Alternatively, participants may may also have chosen not to discuss the negative experiences due to fear, loyalty, or social desirability bias. Therefore, future studies should seek to address this gap to better understand the relationship between healthcare providers and individuals with LF in shaping care pathways.*

22. **does the data speak to other coping mechanism particularly around mental health- what existing support systems do people use, if at all?**

**Response:** Participants typically sought individual-level coping mechanisms, such as praying and engaging in religious activities (Page 21, Line 401). This has been mentioned in Section 3.2.

23. **line 564 says traditional healers remain the primary health care choice while line 357 says only 2 went to traditional healers. please clarify.**

**Response:** We revised the text to resolve the contradiction about traditional healers. Only 2 participants reported visiting them, so we updated the conclusion to state that while traditional healers are often the first point of care in other contexts, our participants more commonly sought public or private facilities.

Please find the following lines on page 34

*In our study, participants sought treatment from different levels of formal and informal healthcare facilities to find cures for their chronic pain and complications. However, other studies have found that traditional healers remain the primary healthcare choice for many people with LF in Bangladesh (16).*

24. **line 575 is a separate section from previous paragraph. Make it a clear break and indicate they are study limitations.**

**Response:** Thank you for your suggestions. We indicated the study limitation section with a clear heading (page 38, Line 743).

25. **a large part of the results was on solutions and this should be addressed in the discussion.**

**Response**: Thanks for the suggestion. We updated the discussion to include the solutions presented in the results section. Please find the following lines in the discussion section (page 32):,

*Participants highlighted a range of needs for better support, including individual, familial, community, and system-level assistance. Firstly, financial aid is provided to those unable to work in traditional sectors, along with job opportunities for their children, emphasising the importance of self-sufficiency and empowerment. Similar findings have been reported in a Sri Lankan study, which demonstrated the effectiveness of providing direct financial support to affected families through the Sri Lankan Samurdhi poverty alleviation scheme, which offers allowances to designated low-income households (17). Secondly, there is a call for community awareness programs through the effective use of technology, as well as improved healthcare services, including mental health support and specialized care corners in hospitals. A study conducted in Nigeria also emphasised that a thorough understanding of transmission factors at the grassroots level would significantly enhance the effectiveness of LF control strategies (18). In terms of well-being, utilizing psychological peer support was proven effective in India in mitigating stigma and enhancing mental well-being, and can be implemented in practice (19). Moreover, study findings suggest that the parallel implementation of enhanced and post-elimination surveillance in hotspots and new areas is also crucial for effectively addressing the spread of filariasis. Additionally, a study from Malawi suggests that post-elimination efforts should be integrated with other national programs and incorporated into routine health systems (20). In light of these findings, future research should focus on evaluating the effectiveness of integrating these strategies and exploring innovative approaches to enhance community engagement and self-sufficiency in affected populations.*

***Programmatic implications of the findings***

*The present study suggests that concentrated efforts are necessary at both the community and policy levels to design interventions that alleviate morbidity and improve the well-being of individuals affected by LF (Table 3).*

| ***Area*** | ***Constraints/ Barriers*** | ***Recommendations/ Opportunities for Interventions*** |
| --- | --- | --- |
| *1. Challenges of seeking healthcare by the persons with LF* | *1.1. Financial barriers* | *a. Providing direct financial assistance or building a social safety net program to ensure financial support.*  *b. Increasing job or rehabilitation opportunities to safeguard economic independence and create pathways to contribute to the national economy.*  *c. Arranging free-of-cost medical support to reduce out-of-pocket expenditures* |
|  | *1.2 Lack of awareness about the condition and community level stigma* | *a. Organizing mass awareness programme to educate people about LF and its chronic health complications, eventually influencing treatment-seeking behaviour.*  *b. Training formal and informal healthcare providers to endure early case detection* |
|  | *1.3. LF inflicted disability* | *a. increasing easy access to healthcare services through digital media*  *b. Involving community level health care workers to reduce distance barriers* |
| *2. Mental health difficulties* | *2.1. Presence of mental distress* | *a. Ensuring mental health facilities at primary-level care facilities.*  *b. Creating and promoting self-help groups to increase the social activity of persons with LF.*  *c. Capacity building of caregivers and families to enhance a positive family environment.*  *d. Training of people with LF on personal hygiene and self-care activities to ensure physical and mental well-being* |

26. **Reviewer #2: This is a very interesting study and important research to inform programme delivery for LF as well as considerations to tailor education and awareness of LF necessary to improve knowledge, reduce stigma and reduce effects on mental health. The paper demonstrates differences by gender which is important when considering gender specific messaging and co unselling.
    This manuscript can benefit from some copy editing, particularly grammatical errors and standards of scientific writing. The results, discussion and conclusion are well written while the intro and methods feel less coherent and would benefit from copy editing.**

**Response:** Thank you for your response. We revised the introduction and methods sections accordingly.

27. **Reviewer #2: In the abstract, it says "about 28 participants"- why "about" rather than being more definitive on the number? Also there are two types of participants (people with LF and key informants) so when talking about participants it should be clear which of the two groups.**

**Response:** We changed "about 28" to the exact number and clarified the breakdown (20 with LF and 8 stakeholders).

**Reviewer #3:**

28. **The study design chosen is sound and rightly helps address the research objectives which is in line with best practices for qualitative research. The objectives were clearly stated. The authors provided a clear explanation and a sound rationale for the study.**

**Response:** Thanks for your comment.

29. **The description of the population is generally clear, however the 8 NTD stakeholders background could be clearer and specific if they are consultant for LF or NTDs in general, this will provide a better context of their experiences to readers (line 224-226).**

**Response:** Thank you very much. We specified whether each stakeholder was a general NTD consultant or specific to LF. This is reflected in a revised Table 2. Please find the following table in page 12:

| *Sl no:* | *Participants ID* | *Gender* | *Position* | *Field of Expertise* | *Work Experience* |
| --- | --- | --- | --- | --- | --- |
| *1* | *NTD_ST_01* | *Male* | *Technical research consultant, non-profit health research institute* | *NTD and LF* | *13 years* |
| *2* | *NTD_ST_02* | *Female* | *Representative (communicable disease elimination programme) of an international non-government organisation* | *NTD and LF* | *23 years* |
| *3* | *NTD_ST_03* | *Male* | *Physician based at a governmental primary healthcare facility at Panchagarh* | *Health service provider of LF affected individuals* | *7 years* |
| *4* | *NTD_ST_04* | *Male* | *Research Consultant, Directorate General Health Services, Ministry of Health* | *NTD, vector-borne diseases and LF* | *20 years* |
| *5* | *NTD_ST_05* | *Male* | *Researcher (Parasitology), Public Health Research Institution* | *NTD, vector-borne diseases and LF* | *9-10 years* |
| *6* | *NTD_ST_06* | *Male* | *Physician and Researcher (studying vector-borne diseases), Public Health Research Institution* | *NTD, vector-borne diseases and LF* | *8 Years* |
| *7* | *NTD_ST 07* | *Male* | *Community-based health worker, International Non-Profit Organisation* | *LF and NTDs* | *4 years* |
| *8* | *NTD_ST 08* | *Male* | *Physician based at a governmental primary healthcare facility* | *Health service provider of LF-affected individuals* | 12 *years* |

30. **In Table 1, can authors clarify what 'Not clear' means under the 'Affected body part'. (Is it that participants could not describe which body part is/was affected?)**

Response: Thanks for the comment. In Table 1, "Not clear" under "affected body part" referred to instances where the participants did not specify which body part was affected by filariasis. We recalled the participants to identify their affected body part; however, among the 9 cases, we could only reach 4 of them on the call. Since most of them had lived with this condition for 15-20 years, their impairment got better.We included updated informations in Table 1.

31. **Generally the authors have a sound analysis plan given that they adopted the COREQ.**

**Response:** Thank you for your remark.

32. **In the methods the Authors described two main participants for the study. However, the first major theme mention '(1) knowledge of LF among affected individuals and CAREGIVERS. The authors can provide a bit of clarity regarding source of data for caregivers experience. (line 230-231) This theme currently suggest caregiver were probably interviewed.**

**Response:** Thank you for your response. It was an error and we have revised the lines.

33. **Authors could help international readers to understand 'Rickshaw' pulling' by adding ('two- or three-wheeled cart').**

**Response:** Thank you. We have mentioned "three-wheel-cart" for better readability for international readers.

34. **Can authors define what SP means ((NTD_SP_03) in line 271.**

**Response**: SP indicates "Stakeholder Participant". This has been mentioned in the revised manuscript.

35. **On line 361-361 authors mentioned hygiene supplies such as 'antibiotic cream, paracetamol, and ointment' I'm not if these three items have been categorised appropriately. Authors should check and describe these items appropriately**

**Response:** Thank you for your observation. The items have been re-categorised appropriately. Please check page 20.

36. **line 400: 'NTD stakeholders agreed about people being forced to prioritise economic survival over their well-being'. Can the authors provide details about how they 'Force' to prioritise....or who force them.....Maybe be rephrase..........**

**Response:** Thank you. We have rephrased the sentence. Please find the following line on page 25, line 477:

*"NTD stakeholders agreed that people had no alternative but to prioritise economic survival over well-being."*

37. **line 406.. do authors want to say the '.......inability to stand and walk due to..........'. Check and correct**

**Response:** Thank you, we have corrected line **482**

38. **In relation to stigma did the authors identified the two dimensions (internalised and external stigma?) as self-isolation can also be as a result of internalised stigma which makes affected persons to hide and disassociate themselves from others. If such narrative was found it will be helpful for authors to highlight this nuases in the stigma experiences to inform all-encompassing stigma interventions.**

**Response:** Thank you very much for valuable concern. We have now clarified the internalised and external stigma in the result (page 17) and discussion section (page 33). Please find the revision in lines 637-649 in the discussion section.

39. **Line 501-502 Is LF medications not already free in Bangladesh? (This is a rhetoric question)**

**Response:** Thank you for addressing this issue. Yes, the medications are free. The Government of Bangladesh provided medications free of cost through the MDA program; however, participants were concerned about the inconsistent supply of free medicines and supplies, as well as the fact that these supplies were insufficient to meet their long-term needs. Additionally, when the free supplies ran out or required specialised care, participants had to incur out-of-pocket expenses to seek support from tertiary and private healthcare facilities for consultations, additional medications, painkillers, and hygiene supplies. We added this part in the result section on page 30, line 586.

*Some participants indicated they needed free medicines for the management of their acute infectious attacks. Although the Government of Bangladesh provided medications free of cost through the MDA program, participants were concerned about the inconsistent supply of free medicines and supplies, as well as the fact that these were insufficient to meet their long-term needs. Additionally, when the free supplies ran out or required specialised care, participants had to incur out-of-pocket expenses to seek support from tertiary and private healthcare facilities for consultations, additional medications, painkillers, and hygiene supplies. One of them mentioned,*

*I believe that if the medicines were provided free of charge, this would help us immensely in adhering to our medication regimen. (female, age 56)*

40. **Reviewer #3: The author's conclusion supports the data analysed in this manuscripts. The authors also described potential biases in the processes as limitations how also provided information on the effort made to address such limitations in order not to affect the interpretation of the findings. The authors have also provided a sound implication of the study which contribute to the knowledge gaps in the experiences of people affected with LF and NTD in general. Generally the findings from this research are relevant for planning for public health interventions for addressing stigma and poor health seeking behaviour.**

**Response:** Thank you

41. **Reviewer #3: The message articulated in this manuscript is sound and clear which makes it easy to follow. The use of clear section headings makes the reading and reviewing easy to navigate. While these broad thematic findings are not new in relation to what we already know with the experiences of many skin NTDs, the authors have added a contextual narratives to the literature to expand the knowledge in the field. I highly recommend it acceptance for publication.**

**Response:** We appreciate your insights.

1.	Rahman KMM, Laskar M, Basu BK, Chowdhury S. Treatment seeking behavior of filariasis patients in a selected endemic area of Bangladesh. Mediscope. 2015;2:5-15.

2.	Ramaiah KD, Kumar KN, Ramu K. Knowledge and beliefs about transmission, prevention and control of lymphatic filariasis in rural areas of south India. Trop Med Int Health. 1996;1(4):433-8.

3.	Ali O, Mengiste A, Semrau M, Tesfaye A, Fekadu A, Davey G. The impact of podoconiosis, lymphatic filariasis, and leprosy on disability and mental well-being: A systematic review. PLoS Negl Trop Dis. 2021;15(7):e0009492.

4.	Person B, Bartholomew LK, Gyapong M, Addiss DG, van den Borne B. Health-related stigma among women with lymphatic filariasis from the Dominican Republic and Ghana. Social Science & Medicine. 2009;68(1):30-8.

5.	Zeldenryk LM, Gray M, Speare R, Gordon S, Melrose W. The Emerging Story of Disability Associated with Lymphatic Filariasis: A Critical Review. PLOS Neglected Tropical Diseases. 2011;5(12):e1366.

6.	Wijesinghe R, Wickremasinghe R, Ekanayake S, Perera M. Treatment-Seeking Behavior and Treatment Practices of Lymphatic Filariasis Patients With Lymphoedema in the Colombo District, Sri Lanka. Asia-Pacific journal of public health / Asia-Pacific Academic Consortium for Public Health. 2008;20:129-38.

7.	Perera M, Whitehead M, Molyneux D, Weerasooriya M, Gunatilleke G. Neglected Patients with a Neglected Disease? A Qualitative Study of Lymphatic Filariasis. PLOS Neglected Tropical Diseases. 2007;1(2):e128.

8.	Bagonza A, Gibson L, Kabiri L, Bezalel C, Narahari SRB, Franks PJ, et al. Exploring barriers to accessing treatment for Lymphatic Filariasis through a socio-ecological lens in Buvuma and Napak districts, Uganda. PLOS Neglected Tropical Diseases. 2025;19(1):e0012747.

9.	Wijesinghe RS, Wickremasinghe AR, Ekanayake S, Perera MS. Physical disability and psychosocial impact due to chronic filarial lymphoedema in Sri Lanka. Filaria J. 2007;6:4.

10.	Gyapong M, Gyapong JO, Adjei S, Vlassoff C, Weiss M. Filariasis in northern Ghana: some cultural beliefs and practices and their implications for disease control. Soc Sci Med. 1996;43(2):235-42.

11.	Coreil J, Mayard G, Louis-Charles J, Addiss D. Filarial elephantiasis among Haitian women: social context and behavioural factors in treatment. Trop Med Int Health. 1998;3(6):467-73.

12.	Ahorlu CK, Dunyo SK, Koram KA, Nkrumah FK, Aagaard-Hansen J, Simonsen PE. Lymphatic filariasis related perceptions and practices on the coast of Ghana: implications for prevention and control. Acta Trop. 1999;73(3):251-61.

13.	Person B, Addiss D, Bartholomew LK, Meijer C, Pou V, Gonzálvez G, et al. "Can it be that god does not remember me": a qualitative study on the psychological distress, suffering, and coping of Dominican women with chronic filarial lymphedema and elephantiasis of the leg. Health Care Women Int. 2008;29(4):349-65.

14.	Dellar R, Ali O, Kinfe M, Tesfaye A, Fekadu A, Davey G, et al. Knowledge, attitudes and practices of health professionals towards people living with lymphoedema caused by lymphatic filariasis, podoconiosis and leprosy in northern Ethiopia. Int Health. 2022;14(5):530-6.

15.	Zeldenryk LM, Gray M, Speare R, Gordon S, Melrose W. The emerging story of disability associated with lymphatic filariasis: a critical review. PLoS Negl Trop Dis. 2011;5(12):e1366.

16.	Haque MI, Chowdhury A, Shahjahan M, Harun MGD. Traditional healing practices in rural Bangladesh: a qualitative investigation. BMC Complement Altern Med. 2018;18(1):62.

17.	Perera M, Whitehead M, Molyneux D, Weerasooriya M, Gunatilleke G. Neglected patients with a neglected disease? A qualitative study of lymphatic filariasis. PLoS Negl Trop Dis. 2007;1(2):e128.

18.	Oluwabiyi B, Oyeyemi OT, Olorunlana A, Omiyeniyi N, Koleosho A. Lymphatic Filariasis in Southwestern Nigerian Rural Communities: A Cross-sectional Survey of the Knowledge, Awareness, and Predisposing Factors. Annals of Global Health. 2016;82(5):806-12.

19.	Agarwal A, Nayak PK, van Brakel WH, van Wijk R, Jain A, Broekkamp H, et al. Impact of basic psychological support on stigma and mental well-being of people with disabilities due to leprosy and lymphatic filariasis: a proof-of-concept study. International Health. 2023;15(Supplement_3):iii59-iii69.

20.	Chiphwanya J, Mkwanda S, Kabuluzi S, Mzilahowa T, Ngwira B, Matipula DE, et al. Elimination of lymphatic filariasis as a public health problem in Malawi. PLOS Neglected Tropical Diseases. 2024;18(2):e0011957.
